# Fun on the Farm: Evaluation of a Lesson to Teach Students about the Spread of Infection on School Farm Visits

**DOI:** 10.1371/journal.pone.0075641

**Published:** 2013-10-16

**Authors:** Meredith K. D. Hawking, Donna M. Lecky, Neville Q. Verlander, Cliodna A. M. McNulty

**Affiliations:** 1 Primary Care Unit, Public Health England, Microbiology Department, Gloucestershire Royal Hospital, Gloucester, United Kingdom; 2 Statistics, Modelling and Economics Department, Public Health England Centre for Infections, London, United Kingdom; The Scripps Research Institute Scripps Florida, United States of America

## Abstract

**Background:**

School visits to farms are a positive educational experience but pose risks due to the spread of zoonotic infections. A lesson plan to raise awareness about microbes on the farm and preventative behaviours was developed in response to the Griffin Investigation into the *E. coli* outbreak associated with Godstone Farm in 2009. This study evaluated the effectiveness of the delivery of the lesson plan in increasing knowledge about the spread of infection on the farm, amongst school students.

**Methods:**

Two hundred and twenty-five 9–11 year old students from seven junior schools in England participated. Two hundred and ten students filled in identical questionnaires covering microbes, hand hygiene, and farm hygiene before and after the lesson. Statistical analysis assessed knowledge change using difference in percentage correct answers.

**Results:**

Significant knowledge improvement was observed for all sections. In the ‘Farm Hygiene’ section, girls and boys demonstrated 18% (*p*<0.001) and 11% (*p*<0.001) improvement, respectively (girls vs. boys *p*<0.004). As girls had lower baseline knowledge the greater percentage improvement resulted in similar post intervention knowledge scores between genders (girls 80%, boys 83%).

**Conclusions:**

The lesson plan was successful at increasing awareness of microbes on the farm and infection prevention measures and should be used by teachers in preparation for a farm visit.

## Introduction

School visits to a farm attraction can be a positive, educational and entertaining experience, enabling students to learn about animal husbandry and food production. However, farm visits can be a source of infection. Farm animals that appear to be healthy can act as a reservoir for harmful microorganisms [1], [2]. Additionally, infectious agents can exist in the farm environment for prolonged periods of time, in soil and on surfaces such as fences and troughs [2], [3], [4], and can contaminate water supplies [5], [6], [7]. In a study of public farms in the Netherlands, researchers found that 64.9% of environmental samples from petting farms were positive for *Escherichia coli* O157, *Campylobacter* or *Salmonella* during the summer months [8]. In an analysis of English working cattle farms, 39% had at least one animal that tested positive for *E. coli* O157 [9].

An international systematic review of school gastro-intestinal outbreaks suggested that 11% of outbreaks originated through animal contact [10]. Close pupil contact in schools facilitates transmission of infectious disease and can also lead to secondary spread into the community [10]. In the largest UK outbreak of *E. coli* associated with farms involving 93 cases, 82% were children under ten years old, whilst nearly half of the visitors to the farm were under 12 years of age [11]. Younger children were more seriously affected.

Despite the health risks, farm visits remain a popular and integral part of childhood education. A recent survey indicated that 80% of primary aged children visited a farm in the last three years and 36% of 7–11 year olds visited with their school [12]. Direct animal contact, such as petting or feeding lambs and goat kids are popular aspects of farm attractions. Shedding of harmful organisms may be higher in these young animals [2], [13], [14]. Common childhood behaviours such as nail biting, thumb sucking, eating, and contact with faeces are risk factors for contracting infection on farms [15], [16]. The Griffin Investigation into the 2009 *E. coli* outbreak acknowledged that there is a low level of awareness about the risk of infection amongst farm visitors and recommended that public education should be reinforced before and during farm visits [11].

e-Bug is a free pan-European educational resource for junior (7–11 years) and senior (12–15 years) schools covering microbes, and the spread, prevention and treatment of infection [17]. Previous research has shown that interactive lesson plans on hygiene and microbiology enable students to retain knowledge beyond the classroom [18], [19]. This paper reports on the delivery of an e-Bug farm hygiene lesson plan focussing on reducing infections following farm visits by school children. We aimed to determine if the delivery of the lesson plan increased student awareness of infection risks and how they can be prevented.

## Methods

During 2010–2011 the e-Bug team in collaboration with Farming and Countryside Education (FACE) created a free interactive lesson plan for teachers on microbes and hygiene on the farm, with web based supporting student resources [File S1]. We planned a lesson to be used before a school visit to an educational farm attraction, which meets the recommendations of the independent investigation into the 2009 Godstone Farm outbreak [11]. Preventative measures to avoid infection on the farm were used to determine the aims of the lesson plan. A focus group with representatives from FACE and farmers who host educational farm visits informed a draft lesson plan which was qualitatively evaluated by a teacher focus group. The final modified lesson plan, aimed at Key Stage 2 (9–11 years) students, consisted of three sections: an interactive class presentation, microbe and animal ‘social networking’ cards and a board game [File S1]. The lesson objectives were then used to formulate statements for the student knowledge questionnaire [File S2].

Using a convenience sample, schools were invited to participate in the study through an article in the FACE newsletter, the e-Bug website and flyers at educational conferences. Minimum sample size was calculated to be 204 key stage two students for power to detect 10% knowledge improvement at the 5% significant level.

The intervention was the lesson given by the students' normal class teacher. Teachers could adapt the lesson plan as they would in a normal classroom situation and all teachers reported doing so. The lesson duration ranged between 1–2 hours depending on the teacher adaptations. Teachers filled out a qualitative questionnaire and schools questionnaire which included background information such as teacher knowledge of the topic and teaching experience. Teachers ran the lesson purely as a classroom exercise, without a farm visit, although one school had a chicken run on site. Teachers were asked to use exam conditions in the classroom to ensure students filled out the questionnaire independently. Students completed the identical questionnaire immediately prior to and following intervention by selecting ‘true’, ‘false’ and ‘don't know’ for each statement [File S2]. The questionnaire contained 29 statements divided into three topics: ‘introduction to microbes’, ‘hand hygiene’ and ‘farm hygiene’ (table 1). Correct ‘true’ and correct ‘false’ statements were included to increase questionnaire validity. The questionnaire style has been used previously during an evaluation of activities undertaken in England, France and the Czech Republic [19].

**Table 1:** Student questionnaire used before and after the farm hygiene lesson.

Section	Question		Correct Response
***Introduction to Microbes***	If you cannot see a microbe it is not there		FALSE
	All bacteria are harmful		FALSE
	Bacteria and Viruses are the same		FALSE
	Fungi are microbes		TRUE
	Microbes are found:	in boiling water	FALSE
		in our mouths	TRUE
		on our hands	TRUE
		on animals	TRUE
***Hand Hygiene***	Bad microbes can spread when you touch someone's hands		TRUE
	When visiting a farm people should wash their hands:	before eating	TRUE
		After petting the animal	TRUE
		After eating	FALSE
		after touching the crops	FALSE
	If people wash their hands, they are less likely to be ill		TRUE
	Washing with soap **and** water removes more microbes than water alone		TRUE
	Washing hands with alcohol gel/wipes will remove all bad microbes on the farm		FALSE
	Using alcohol hand gel is better than washing hands with hot running water and soap		FALSE
***Farm Hygiene***	At the farm, microbes are found:	On cows	TRUE
		On gates	TRUE
		In the grass	TRUE
		On your wellie boots	TRUE
	Some microbes can help crops to grow		TRUE
	There are more useful microbes on the farm than harmful ones		TRUE
	There is no need to wash your hands after stroking a farm animal		FALSE
	Washing hands is the best way to stop the spread of harmful microbes		TRUE
	You cannot pick up bad microbes from kissing or hugging an animal on the farm		FALSE
	It is OK to eat your sweets while walking around a farm		FALSE
	We should wash our wellie boots before we leave the farm		TRUE
	We should eat our packed lunch at the farm picnic tables		TRUE

### Ethics Statement

Informed written consent was obtained from all schools that took part prior to enrolment. Teachers gave consent for students to be involved as no student identifiers were used and the questionnaires were used to evaluate the learning outcomes of the sessions as they would in everyday lessons, therefore parental consent forms were not required for schools to be involved in the evaluation. Schools could notify parents that children were taking part at their own discretion. As it was an educational evaluation and no NHS staff or patients were involved formal Research Ethics Committee review through the National Research Ethics Service was unnecessary. This is in accordance with the National Research Ethics Service ‘defining research’ guidelines, which characterise the study as ‘service evaluation’ for the purposes of ethical review. These guidelines can be accessed at the following link: http://www.nres.nhs.uk/applications/is-your-project-research/. All data was anonymised and stored and handled according to the Data Protection Act 1998 and Caldicott 1999 regulations. There was no financial reward for schools that took part.

### Statistical analysis

Responses were coded and double entered using EpiData 3.1 for accuracy. Mixed effects linear regression was performed on section percentage correct, starting with a full model with a two-way interaction between the fixed effects of teaching phase (before and after) and gender (male and female), with pupil as the random effect. Records with unspecified gender were treated as missing and excluded when analysis involved gender. If the interaction was not significant (p>0.05), as determined by Wald testing, it was dropped. For those sections where there was a significant interaction, the percentage improvement was calculated for each gender and the p-value for the interaction quoted. Where there were no significant interactions, sign-rank test p-values and the overall percentage improvement were calculated. A similar approach was adopted for question-specific percentage correct, except that the mixed effects linear regression and sign-rank tests were replaced by mixed effects logistic regression (where the outcome was a binary variable taking values right and wrong) and McNemar's test for paired data, respectively. Where there were significant interactions, percentage change and 95% CI were obtained for the question specific questions by stratification of the gender variable. All analyses were performed using STATA version 12.

## Results

Eleven schools initially agreed to participate; this included 16 classes and 415 children in seven rural and four town localities in five regions across England (North West, East Midlands, West Midlands, East of England, and South East). Four schools did not complete the lesson nor return the questionnaires due to time restraints as a result of an Ofsted inspection or change in school staffing (teachers moving posts), or personal circumstances of the teachers who enrolled in the study. Amongst the seven schools that returned questionnaires, questionnaire return rate from students was 93%. Therefore the final sample for analysis included 210 junior (key stage two, 9–11 years) students from seven primary schools from five regions. Of the seven primary schools analysed, four were classified as ‘rural’, three classified as ‘town’ and none classified as ‘city’ by the researchers and teachers. There were no significant differences in section answers between rural and town based students. Students in two schools (n = 48) had been taught about microbes as part of other topics in the previous school year and the remaining students had not been taught the topics before.

### Introduction to Microbes

Overall post intervention, the percentage of questions answered correctly for the ‘introduction to microbes’ section were significantly greater than the percentage pre-intervention scores for both girls and boys: girls increased their knowledge by 21% (*p*<0.001) from 58% correct questions at baseline, whilst boys increased knowledge by 14% (*p*<0.001) from 69% correct questions before (table 2). All statements in this topic showed significant improvement, with ‘microbes are found on animals’ improved from 81.1% correct before by 16.4% (10.1 to 22.7, *p*<0.001) to 97.5% correct answers after intervention (table 3). Correct responses to ‘if you cannot see a microbe it is not there’ improved by 20.0% (13.7 to 26.3, *p*<0.001) from 73.8% to 93.8% after the lesson was undertaken.

**Table 2:** Results: percentage of all questions in each section correct before and after lesson, by questionnaire section.

Questionnaire Section		Correct answers pre-intervention (%)	Correct answers post-intervention (%)	Improvement score (%)	Pre vs Post P value	Difference between genders P value
***Introduction to microbes:***	**Male**	69	83	14	<0.001	0.03
	**Female**	58	79	21	<0.001	
***Farm hygiene:***	**Male**	72	83	11	<0.001	0.004
	**Female**	62	80	18	<0.001	
***Hand hygiene***		67	71	4	<0.001	

**Table 3:** Results: percentage of correct answers before and percentage improvement for each statement in the evaluation questionnaire.

Section	Question		Correct answers before (% correct)	Improvement score (% improvement)	95% CI	*P* value
***Introduction to Microbes***	If you cannot see a microbe it is not there		155 (73.8)	197 (20.0)	13.7, 26.3	<0.001
	All bacteria are harmful		162 (77.5)	187 (12.0)	5.6, 18.4	<0.001
	Bacteria and Viruses are the same		126 (60.6)	147 (10.1)	2.4, 17.8	0.007
	Fungi are microbes		68 (33.3)	127 (28.9)	20.6, 37.2	<0.001
	Microbes are found:	in boiling water	89 (47.3)	108 (10.1)	2.0, 18.2	0.01
		in our mouths	110 (57.3)	163 (27.6)	19.6, 35.7	<0.001
		on our hands	152 (77.9)	173 (10.8)	3.8, 17.7	0.001
		on animals	163 (81.1)	196 (16.4)	10.1, 22.7	<0.001
***Farm Hygiene***	At the farm, microbes are found:	On cows	166 (80.2)	201 (16.9)	10.5, 23.3	<0.001
		On gates	110 (55.3)	180 (35.2)	27.5, 42.9	<0.001
		In the grass	110 (55.0)	172 (31.0)	22.9, 39.1	<0.001
		On your wellie boots	124 (62.3)	176 (26.1)	18.8, 33.5	<0.001
	Some microbes can help crops to grow		109 (52.9)	173 (31.1)	22.8, 39.3	<0.001
	There are more useful microbes on the farm than harmful ones		53 (25.5)	62 (4.3)	−4.1, 12.7	0.3
	There is no need to wash your hands after stroking a farm animal		194 (95.1)	192 (−1.0)	5.3, 3.4	0.6
	Washing hands is the best way to stop the spread of harmful microbes		182 (87.5)	187 (2.4)	−3.1, 7.9	0.4
	You cannot pick up bad microbes from kissing or hugging an animal on the farm		171 (81.4)	186 (7.1)	0.9, 13.4	0.02
	It is OK to eat your sweets while walking around a farm		133 (64.3)	159 (12.6)	5.5, 19.7	<0.001
	We should wash our wellie boots before we leave the farm		133 (63.6)	164 (14.8)	7.6, 22.1	<0.001
	We should eat our packed lunch at the farm picnic tables		164 (78.8)	157 (−3.4)	−10.0, 3.3	0.3
***Hand Hygiene***	Bad microbes can spread when you touch someone's hands*					
	Male		84 (80.0)	86 (1.9)	−7.0, 10.8	0.11
	Female		69 (71.1)	86 (17.5)	7.9, 27.1	0.001
	When visiting a farm people should wash their hands:	before eating	177 (88.9)	174 (−1.5)	−6.7, 3.7	0.5
		After petting the animal	190 (96.4)	194 (2.3)	−1.3, 5.3	0.16
		After eating	80 (43.0)	78 (−1.1)	−7.8, 5.6	0.7
		after touching the crops	38 (20.1)	26 (−6.3)	−11.9, −0.8	0.01
	If people wash their hands, they are less likely to be ill		187 (90.3)	194 (3.4)	−0.2, 7.0	0.03
	Washing with soap **and** water removes more microbes than water alone		182 (87.5)	189 (3.4)	−2.2, 8.9	0.19
	Washing hands with alcohol gel/wipes will remove all bad microbes on the farm		78 (38.2)	125 (23.0)	15.1, 31.0	<0.001
	Using alcohol hand gel is better than washing hands with hot running water and soap		110 (53.4)	130 (9.7)	1.3, 18.2	0.02

* Significant difference between genders, *p* = 0.02.

### Farm Hygiene

In the farm hygiene section, there was overall significant improvement in the percentage of correct answers from baseline (baseline scores for girls: 62%, boys: 72%) of 11% (*p*<0.001) in boys, and 18% (*p*<0.001) in girls (table 2). In particular, statements relating to awareness of microbes on the farm showed high knowledge improvement, such as: at the farm microbes are found: ‘on cows’ 16.9% (10.5 to 23.3, *p*<0.001), ‘on gates’ 35.2% (27.5 to 42.9, *p*<0.001), ‘in the grass’ 31.0% (22.9 to 39.1, *p*<0.001), and ‘on your wellie boots’ 26.1% (18.8 to 33.5, *p*<0.001), and ‘some microbes can help crops to grow’ 31.1% (22.8 to 39.3 *p*<0.001) (table 3). Some statements about health related behaviours showed significant improvement in correct answers, such as ‘we should wash our wellie boots before we leave the farm’ 14.8% (7.6 to 22.1, *p*<0.001) and ‘it is ok to eat your sweets while walking around the farm’ 12.6% (5.5 to 19.7, *p*<0.001). Questions related to hand washing showed the lowest levels of improvement, e.g. ‘there is no need to wash your hands after stroking a farm animal’ −1.0% (−5.3 to 3.4, *p* = 0.6) and ‘washing hands is the best way to stop the spread of harmful microbes’. 2.4% (−3.1 to 7.9, *p* = 0.4).

### Hand Hygiene Section

Within the hand hygiene section there was a small, but significant 4% (*p*<0.001) increase in overall questions correct (table 2). Knowledge relating to the use of hand gels improved, for example ‘washing hands with alcohol gel/wipes will remove all bad microbes on the farm’ 23.0% (15.1 to 31.0 *p*<0.001), and ‘using alcohol hand gel is better than washing hands with hot running water and soap’ 9.7% (1.3 to 18.2 *p* = 0.02) (table 3).

### Gender

Girls showed significantly greater improvement in percentage of answers correct than boys for the ‘farm hygiene’ and ‘introduction to microbe’ topics (*p*<0.004), but correct answers before intervention were lower in girls than in boys for both sections. Consequently, the after questionnaires for both genders demonstrated a similar percentage of correct answers (farm hygiene section: girls 80%, boys 83%). There was no significant difference between the gender specific knowledge change in the hand hygiene section.

## Discussion

### Main Findings

This is the first free school lesson plan on microbes and the spread of infection to incorporate into farm visits shown to improve knowledge in a formal evaluation. The results show a significant improvement in farm hygiene and microbiology knowledge after intervention, especially relating to statements on awareness of microbes on the farm and preventative measures such as washing footwear before leaving and ‘hand to mouth’ behaviours. High levels of knowledge about hand washing was indicated in the baseline scores, but despite this, improvements in some aspects of hand hygiene knowledge were seen, for instance relating to awareness about the limitations of hand gels and hand wipes on the farm. Post intervention knowledge levels were similar between boys and girls in the study, although results indicated that girls had lower pre-intervention knowledge about microbes and farm hygiene compared to boys. Overall the lesson plan was effective at improving awareness of the spread of infection in a farm setting amongst the target group.

### Other work in this area

A range of informative resources for teachers about the risk of infection on the farm are currently available from a number of sources. These include: fact sheets and guidance for teachers and farmers [20], [21], [22], short activities [23] and a farm health and safety video [24]. However, delivery of the e-Bug farm hygiene lesson has been shown to be an effective way of passing health related knowledge directly to students. The lesson is free, easily accessible and was developed by health professionals in collaboration with farmers, teachers and FACE, and has been evaluated by teachers and students, making it an important resource to help prevent farm-related infection in children and its use should be encouraged in connection with farm visits.

### What this study adds

Our positive results have important implications for the health of young people who experience more serious symptoms related to gastro-intestinal infections contracted on farm visits [25]. Improved knowledge around preventative behaviours, such as not eating as you walk around the farm, is important because ‘hand to mouth’ behaviours are a particular risk for acquiring farm related infection [15], [16]. Infections originating in a farm setting can result in secondary spread in the home [25] for example there were 13 secondary cases associated with the 2009 *E. coli* outbreak [11]. The increased knowledge of the benefits of washing footwear in our study should help to reduce the risk of contracting farm related infections and reduce secondary spread in the home if there are sufficient facilities for boot washing on farm premises or in the home.

Farm visitors should wash hands with soap to avoid infections on the farm [11], [26]. Interventions focusing on improving hand washing can reduce absenteeism from gastrointestinal diseases in school settings [27] and a Cochrane review demonstrated hand washing interventions aimed at children (<15 yrs) can reduce diarrhoeal illness by around 39% in high income countries [28]. In our study knowledge on hand washing after animal contact did not significantly improve, however baseline knowledge was very high for these questions – for example ‘there is no need to wash your hands after stroking a farm animal’ was answered correctly by 95% of students at baseline. Other research in this area has shown that 98% of primary aged children knew that they should wash their hands after contact with an animal [29]. Concordantly, we also found high baseline knowledge in the hand hygiene section (90.2 to 95.6%), only allowing for a small but significant increase (4%, *p*<0.001) in overall knowledge. Despite widespread awareness about hand washing, children are more likely to self report hand washing behaviour if they understand the reasons for doing so [29]. Improvement in knowledge about microbes on the farm may therefore be a successful influence on positive health-related behaviour in primary aged children.

Significant gender differences were observed in our study for the ‘introduction to microbes’ and ‘farm hygiene’ sections. Girls initially had lower baseline scores than boys but demonstrated greater improvement resulting in similar post intervention knowledge levels between the genders. This is salient, because some research suggests that female sex can be a risk factor for having antibodies to *E. coli* O157, which may in turn be a surrogate marker for behaviours that increase risk for contracting *E. coli* infection [30], for instance hugging and kissing animals. However, this may not be true for other pathogens, for instance campylobacteriosis, where higher rates can be seen in young males [31]. In this study knowledge in this area was improved, for instance ‘you cannot pick up bad microbes from kissing or hugging an animal on the farm’ was increased by 7.1% (0.9 to 13.4, *p* = 0.02). Animals have been described as the most popular aspect of farm visits, especially for girls, amongst whom 65% reported animals as their favourite part of a farm visit [12]. The similar gender specific post intervention scores indicated in the results demonstrate the suitability of the lesson plan for addressing gender specific risk of infection on the farm.

### Limitations of this study

The study group was convenience sampled through the e-Bug and FACE websites and associated newsletters, potentially introducing non-random sampling bias. Due to this, rural schools and schools located in towns were included; however no schools from inner cities were recruited into the study. This may reduce the generalisability of the results to these inner city schools. Although teachers self selected, the students themselves did not choose to opt in or out of the study. Whilst teachers may have had personal characteristics and greater interest in the subject area that pre-determined their wish to participate in the study, this would be less likely for students. Furthermore four out of the seven teachers in the study reported that they had little (as opposed to none or extensive) knowledge of the subject area so this may not be an important drawback.

The response rate from recruited schools was 64% - four schools out of eleven failed to complete the evaluation and return the questionnaires, introducing potential non-response bias. However, schools that failed to return questionnaires gave reasons relating to time constraints and staffing issues, suggesting that non return was not due to lack of enthusiasm for the subject that would differentiate them from the responding schools. Equally, non response in educational studies involving schools may occur as teachers have little time to devote to activities outside of their normal school duties.

We measured knowledge change, rather than behaviour change. The extent to which knowledge improvement can affect behaviour has be questioned - in a study of primary students, 98% knew they should wash hands after contact with an animal but only 79% self reported ‘always’ doing so [29]. However, research has shown that awareness of the risk of infection in a farm environment can be protective against infection [16]. Furthermore, realistic learning conditions such as simulations or life experience may be more effective than standard lessons at helping children learn health-related behaviours [32], [33]. Demonstrating change in knowledge with this lesson is a very important first step, but future research should appraise the effectiveness of the combination of a lesson followed by real experience of a farm visit for consolidating knowledge and helping children learn health-related behaviours. Knowledge retention was not tested, which may be a disadvantage of the study. However, the lesson plan was designed for use in preparation before going on a school visit to a farm, so this limitation may not reduce the applicability of the results, as in these circumstances the lesson plan would be followed by the farm visit with little or no delay. The lesson plan in its entirety or parts of it could also be modified for use by farmers at the farm education venue so that improved awareness of spread of infection on the farm could be utilised immediately by visitors. Using identical before and after questionnaires may have introduced bias as students can become ‘attuned’ to questions through the process of completing the questionnaire and undertaking the lesson. Finally, schools delivered the lesson plan in ‘real’ conditions to reduce potential experimental bias – teachers gave the lesson as they would normally, including adapting the lesson plan to the needs of their students. We were unable to evaluate whether variation in lesson duration had an impact on knowledge, however using realistic conditions in this study may increase the applicability of the results to school settings as schools have different amounts of time to devote to lesson topics in day to day practice.

## Supporting Information

File S1 e-Bug Fun on the Farm Lesson Plan.
(PDF)

File S2 Pre- and Post- Intervention Student Knowledge Questionnaire.
(PDF)
