# Supplementary material for: Fun on the Farm: Evaluation of a Lesson to Teach Students about the Spread of Infection on School Farm Visits
Source: PLoS One. 2013 Oct 16;8(10):e75641. doi: 10.1371/journal.pone.0075641 (PMC3797722; doi:10.1371/journal.pone.0075641)

e-Bug

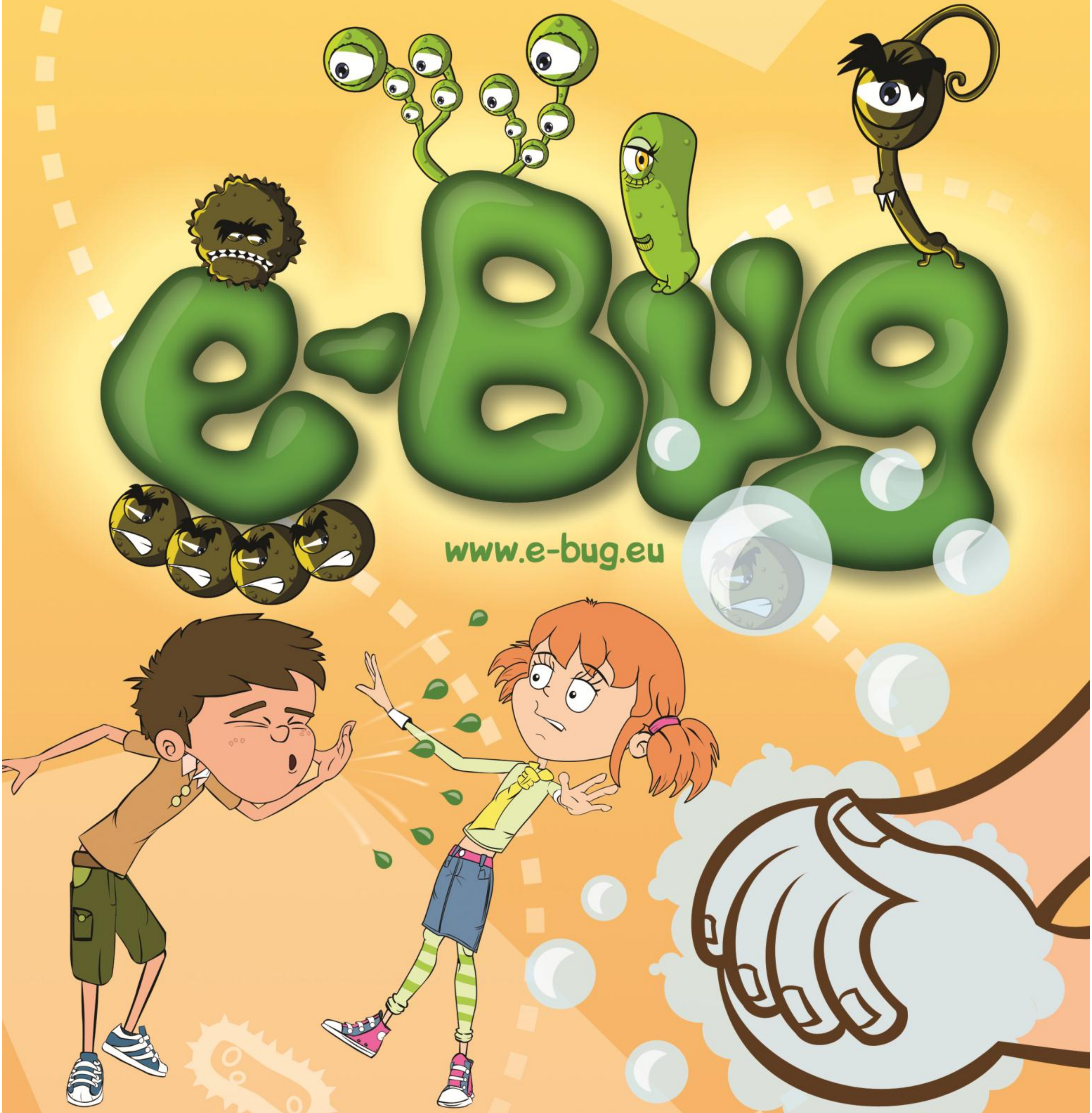

department for
children, schools and families

Key Stage 2 / Science
Years 5 & 6

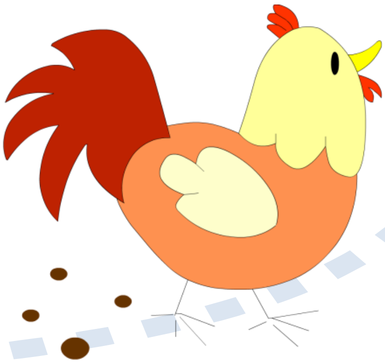

National Curriculum Links

Key Stage 2
Sc1: 1a, 1b, 2a, 2b, 2c, 2d, 2e, 2f, 2g, 2h, 2i, 2j, 2k, 2l
Sc2: 2g, 5f

Unit of Study
Unit 6 – Micro-organisms

Estimated Teaching Time
50 minutes

2.4 Spread of Infection Farm Hygiene

The Spread of Infection section aims to teach students how poor hand hygiene, respiratory hygiene and improper care with food can lead to the spread of microbes and disease.

Section 2.4, Farm Hygiene, teaches students that the farm environment is home to both useful and harmful microbes. Students learn through group discussion, 'social networking', and a farm journey board game, the risks and benefits of various farm microbes.

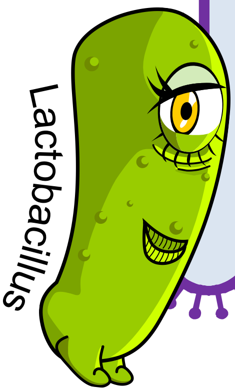

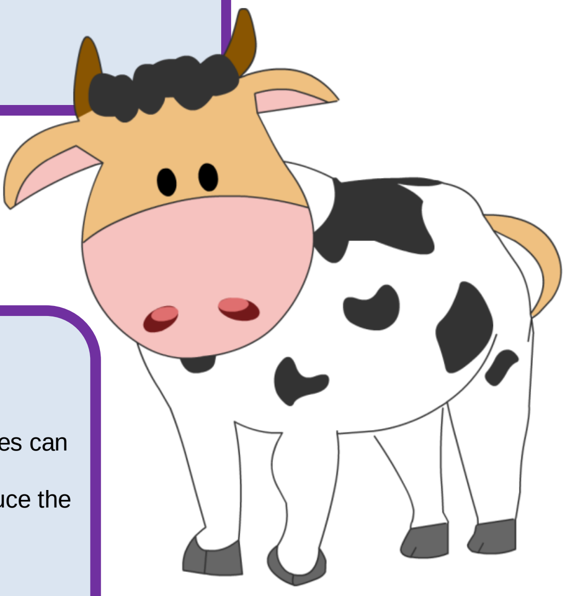

Learning Outcomes

All students will learn that:

- Useful microbes on the farm help the farmer with food production
- Harmful microbes can be found on the farm and that these microbes can spread to humans
- By washing our hands and following some basic rules we can reduce the chance of picking up an infection on the farm

More able students will learn:

- Where specific harmful microbes can be found on the farm
- Where specific useful microbes can be found on the farm and how they are used in food production

2.4 Spread of Infection
Farm Hygiene

Key Words
Farm
Hygiene
E. coli
Salmonella
Campylobacter
Useful microbes
Rhizobia
Thermophiles
Lactobacilli

Materials Required

Per student group
- ☐ Copy of SW 2 - SW 8
- ☐ Farm Journey Game: board, counters, dice and cards

Per Class
- ☐ Copy of PowerPoint slides or SW 1 for each student

Available Web Resources

- A demonstration of this activity
- PowerPoint slides for main activity
- Interactive 'Farm Fun' game on Junior website to reinforce message of this lesson
- A film clip on Junior website showing the recommended method of hand washing with soap

Alternative Suggestion

If PowerPoint is not suitable, PDFs of each slide can be downloaded and either copied on to acetates for an overhead or blown up for use as visual aids.

Background Information

The farm environment is a place of fun and education for people of all ages. It is essential however, to be aware of the microbes associated with the farm environment, some of which can be harmful to humans, to ensure that farm visits are enjoyable and safe. Farm animals, even those that appear clean and healthy, may carry microbes that are useful and harmless to the animal, but can make us very ill if they get inside our body. *Escherichia coli, Salmonella* and *Campylobacter* are examples of some of the bacteria that can be a cause of infection for all ages, but the symptoms can be particularly serious for young children. These bacteria are normally carried in the animal droppings and as such, may be present anywhere faeces can be found, for example on gates, fences, on the animals face, etc. N.B. it only takes a small number of these bacteria to cause an infection.

Symptoms of each microbial infection can vary but generally include rapid fluid loss from diarrhoea and vomiting and can lead to death in very severe cases.

However there are many more useful microbes on the farm than harmful microbes. These include *Lactobacilli* that ferment silage and turn milk into yogurt; thermophiles that break down decaying plant matter in compost; and *rhizobia* that change atmospheric nitrogen gas into ammonia in the soil. This lesson plan aims to teach students about useful and harmful microbes in the farm environment.

Students will learn some of the simple steps they can take to reduce the risk of picking up an infection on a visit to a farm. They will also learn more about how microbes can spread in different environments.

Some of these simple steps include:

- Washing hands with soap and water after contact with animals and before eating and drinking (alcohol gel or hand wipes will not remove these microbes effectively).
- Avoid kissing or putting your face close to the animal's face; and avoid putting your own hands near your face or in your mouth.
- Eating in the designated picnic areas/ cafe facilities.
- Not eating anything whilst walking around the farm or anything that has dropped on the floor.
- Washing soiled footwear thoroughly and then washing your hands thoroughly with soap and water.

Advance Preparation

Copy SW 2 - SW 8 for each student group (4-5 students per group). Download PowerPoint slides or prepare SW 1 for each student/group
Prepare laminated board game for each group.

2.4 Spread of Infection
Farm Hygiene

Lesson Plan

Introduction

1. Begin the lesson by asking students to identify some of the risks when visiting a farm e.g. tripping on uneven ground, working farm machinery, etc. Then ask if they think there is anything on the farm that could make them ill, e.g. eating the animal food! End the discussion by asking if there could be any harmful microbes on the farm that could make them ill.
2. Explain that **some** farm animals (that appear clean and healthy) may have microbes living on them that can't be seen, and don't cause any harm to the animal, but that can make us ill if we pick them up.
3. Explain that these microbes normally live in animal droppings but that sometimes these droppings get on lots of different things. Ask students to imagine a cow scratching its bottom on a fence, and then scratching its face at the same place on the fence. Where are all the harmful microbes from the droppings now? *On the cows bottom and face but also on the fence*. Explain that when we touch these animals or places the animals have been we could pick up these harmful microbes on our hands, and if these microbes got inside our body, they could cause vomiting and diarrhoea.
4. Tell the students that they are going to learn about some of the harmful microbes at farms and how they can easily protect themselves when they visit a farm and touch the animals. Also tell them that they are going to learn about some useful microbes that can be found on the farm, and how these help in food production.

Main Activity

1. Present the students with each of the animal images (PowerPoint slides or PDFs can be downloaded from the website www.e-bug.eu).
2. For each animal, ask students to answer the question and discuss (teaching points are detailed in TS 4).
3. Then ask the students where on the farm they think there may be microbes that are useful to humans.
4. Ask the class if they have any ideas what these harmful and useful microbes on the farm might be called. Give students SW 1 – SW 6.
5. Ask the students to discuss what they can tell from the profiles about the risks and benefits of the different microbes, and whether they would want to make them their 'friends'. Discuss anything they noticed in the profiles that could help prevent the harmful microbes from spreading to people.

Secondary Activity

This 'fun on the farm' game will highlight precautions needed to prevent the spread of infection on a farm.

1. Group the students into teams of around 6 and give each group a board, counters, dice and pack of cards.
2. The students play the game by rolling the die and moving their counter along the board. If they land on a harmful microbe or useful microbe, the player to their left must pick up a card and ask the question on that card. The used card is then put to the bottom of the pack and the next player rolls. The winner is the player that gets to the end of the board first.

This game can also be played before a school trip to a farm. The scenarios can be linked to what the students will be doing, such as a tractor ride, or played as described above before the farm tour starts.

2.4 Spread of Infection
Farm Hygiene

Lesson Plan

Plenary (after classroom activity OR farm visit)

1. Discuss with students the importance of what they have learnt

   It is important to be aware of harmful microbes that animals may carry and maintain good hygiene in order to protect yourself from becoming ill.

   It is also important to remember that there are many more useful microbes on the farm that help farmers produce food for us every day.

2. Ask the class to pick out some things that they can do to protect themselves if they visit a farm. For example:

   a. *Wash their hands with soap and water after touching the animals and before eating and drinking anything*

   b. *Eat in the designated picnic/ cafe area*

   c. *Do not eat anything that has fallen on the floor*

3. Use this discussion to reinforce the main key health messages:

   - *Farm animals can carry harmful microbes such as Salmonella, E. coli and Campylobacter that can make us unwell.*
   - *Washing hands with soap and water is very important, especially after touching animals and before eating and drinking.*
   - *Animals should not be kissed or touched near the mouth, rear end or feet; and children should avoid touching their faces or mouths whilst on the farm.*
   - *If children do get ill after visiting a farm, parents should contact their GP and they should remain off school for two days after the sickness and diarrhoea symptoms end, to reduce the spread of infection amongst their peers.*

Extension Activity

Visit the Junior student section of the e-Bug website, **www.e-bug.eu**, to access the 'Farm Fun' interactive learning activity. This interactive animation allows students to spot things that the young visitors are doing wrong on the farm, and explains why each action may lead to harm.

2.4 Spread of Infection
Farm Hygiene

Teaching Points

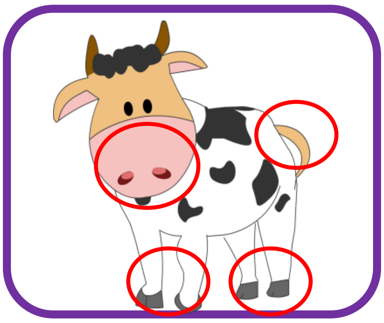

Where on the cow might you find lots of harmful microbes?

Microbes tend to be concentrated in certain areas, such as the mouth, rear end, tail and feet, and students should avoid touching animals in these places, as they are more likely to become infected if they do. E. coli lives in the gut of the animal where it helps the animal digest food, and is therefore more likely to be concentrated in areas on the faecal-oral route and on the ground where droppings have been. Ask the students where they think the best place on the animal would be to stroke or pat (the answer should be the flank, neck or back).

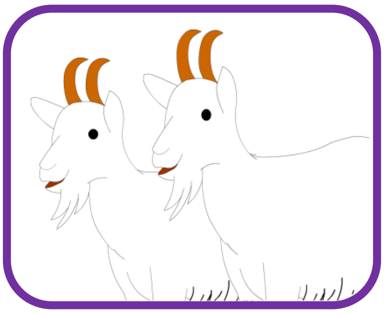

Where on the farm might these goats spread any microbes they carry?

The goats can spread harmful microbes on any ground/ areas on the farm that they walk on, any fences that they can touch, feeding equipment they eat from, or any humans or other animals that they have contact with. And remember that animals carry microbes that they need to keep them healthy, but if these microbes pass to humans they can make us unwell (for example E. coli in the animals gut helps the animal to digest food but if we ingest them they can give us diarrhoea). So we should wash our hands after contact with any animal on the farm, including farm pets like dogs and cats.

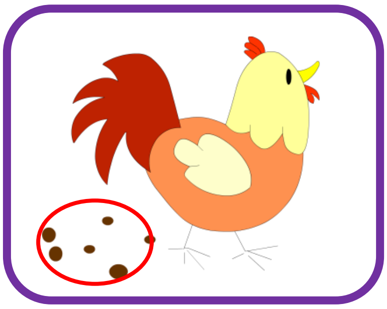

Why must you be careful to wash your hands after holding this chicken?

Chickens are fun to hold and make good pets, but chicken droppings can carry harmful microbes, so you should avoid stepping in the droppings if possible and make sure to wash your hands thoroughly with soap and running water after contact with the chickens.

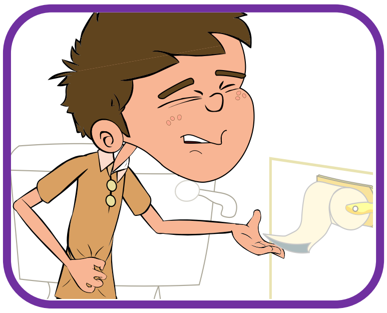

Harry caught a tummy bug after visiting a farm – do you know why?

There are lots of useful and harmful microbes on animals and on surfaces around the farm, and a few of these microbes don't harm the animals but can make us ill! It is important to wash your hands properly to remove any harmful microbes which may be present after contact with animals, and before you eat or drink anything. If you do become ill you should stay off school, drink lots of water and inform your GP that you have been on a farm visit.

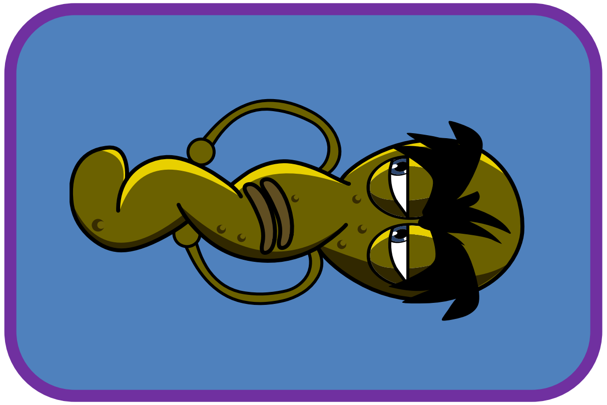
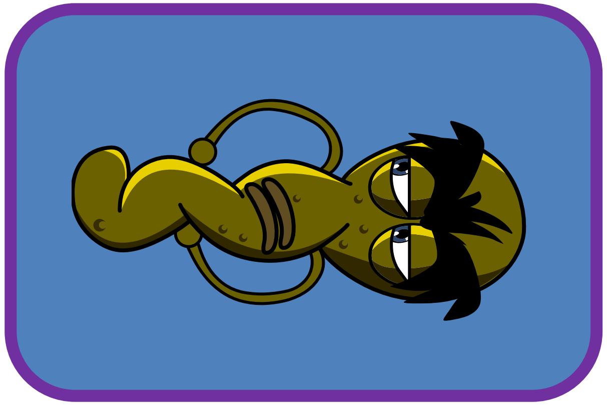
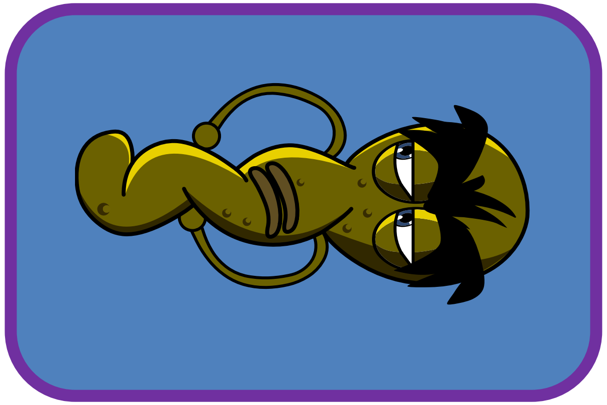
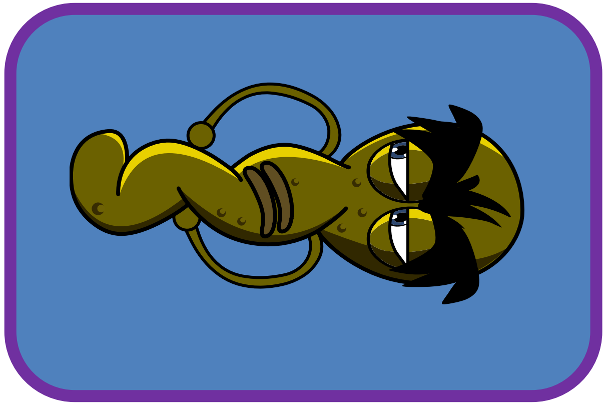
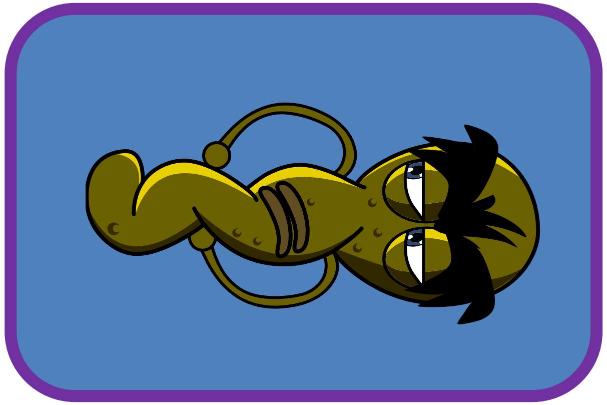
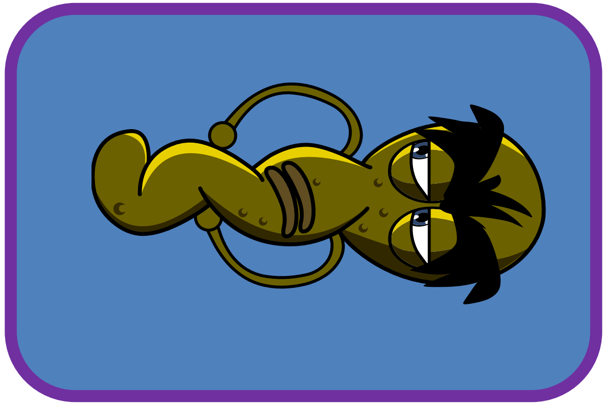
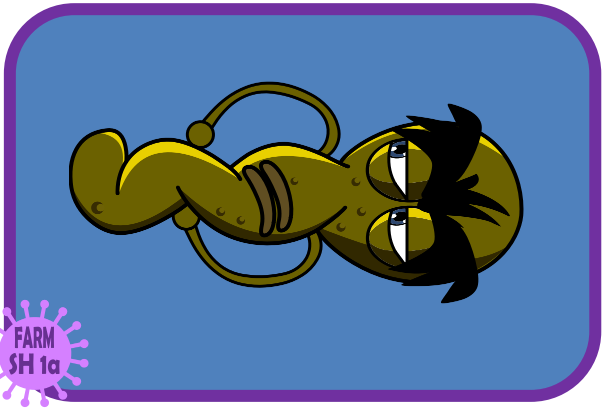
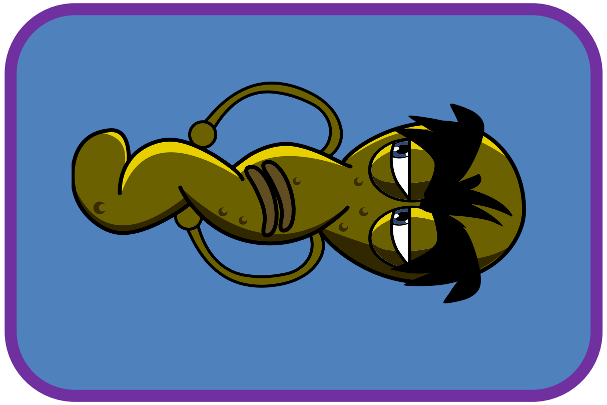
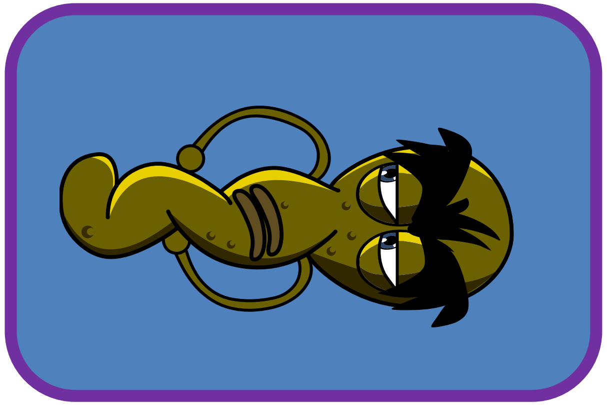

FARM
SH 1a

Move back 3 spaces

But stay where you are if you can answer the question

Why should you not eat sweets in the animal pen?

Harmful microbes can be passed to your mouth when you eat, which can make you unwell

Swap places with the person in last place

But stay where you are if you can answer the question

Why should we wash our hands after playing with animals?

Animals carry harmful microbes that can make you unwell. You can pick these microbes up when you touch them.

Swap places with the person behind you

But stay where you are if you can answer the question

Why should we only eat food where the farmer tells us?

The farmer will tell you to eat in an area where you can wash your hands and where animals aren't allowed, so there will be less chance of picking up harmful microbes.

Move back 5 spaces

But stay where you are if you can answer the question

Why might there be harmful microbes on fences?

Animals carry harmful microbes on their body and can pass these microbes on to the fence when they walk past the fence, scratch their behind on it or lick or slobber on it.

Move back 4 spaces

But stay where you are if you can answer the question

There are only harmful microbes on the farm.
Is this true or false?

False – there are lots of useful microbes on the farm that help the farmer produce food for us to eat.

Move back 1 space for every wrong answer

Name 3 places on the farm where there might be harmful microbes?

Three from: On an animal's body, in the animal's pen, in animal's poo, on a fence, on the ground where animals walk

Move back to space number 2

But stay where you are if you can answer the question

Can you name 1 harmful microbe that you might find on the farm?

Either: *E. Coli*, Salmonella, Campylobacter

Move back 2 spaces

But stay where you are if you can answer the question

The teacher caught you sucking your thumb when you were playing with a pet calf. Why is this a bad thing to do?

Harmful microbes can be passed to your mouth when you suck your thumb, which can make you unwell.

Move back 1 space

But stay where you are if you can answer the question

Using soap and water to wash your hands is better than using hand gel.
Is this true or false?

True, because it removes more harmful microbes.

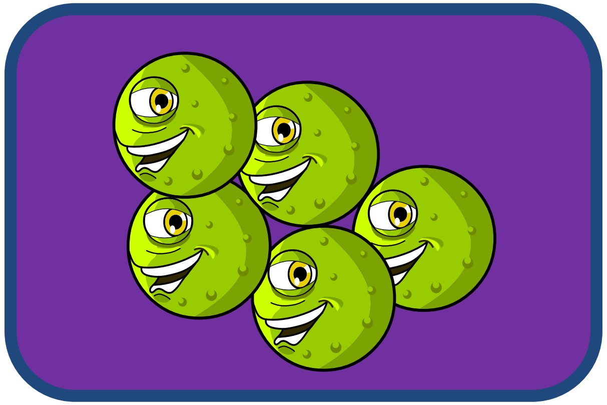
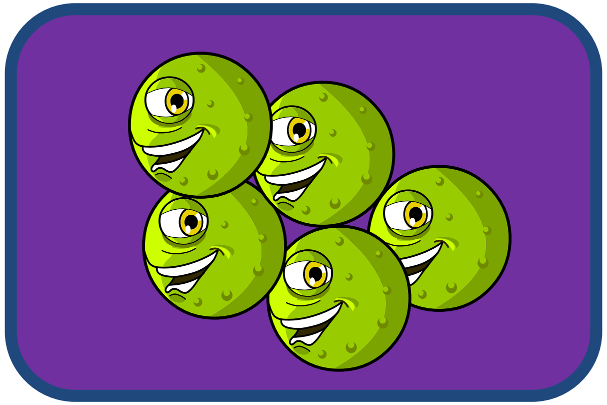
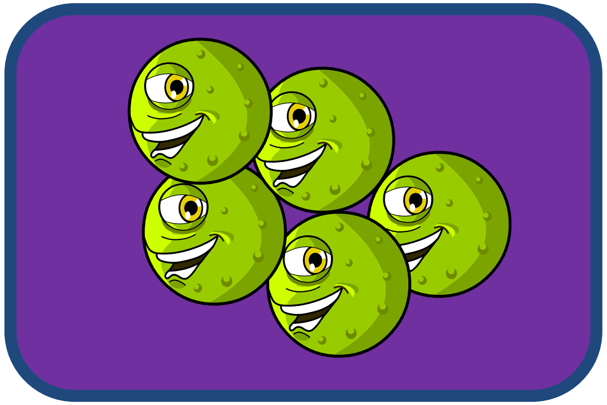
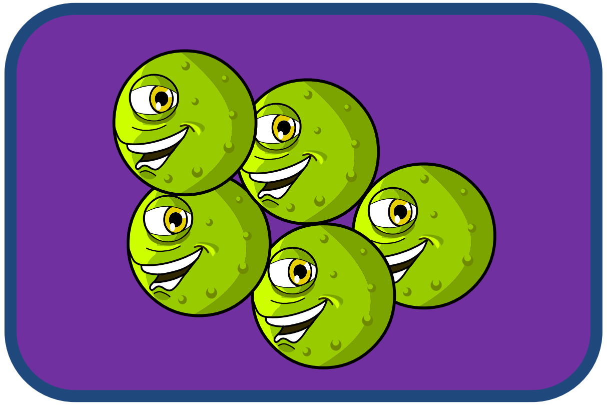
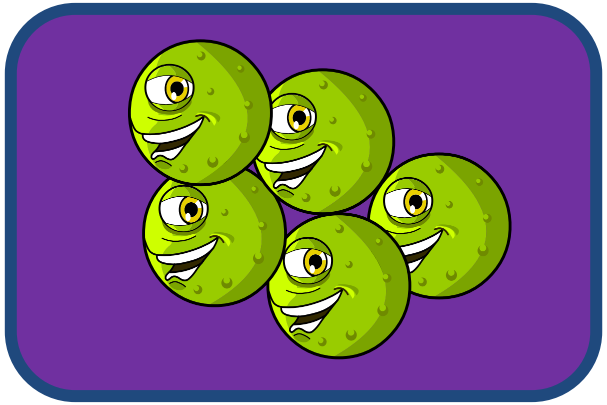
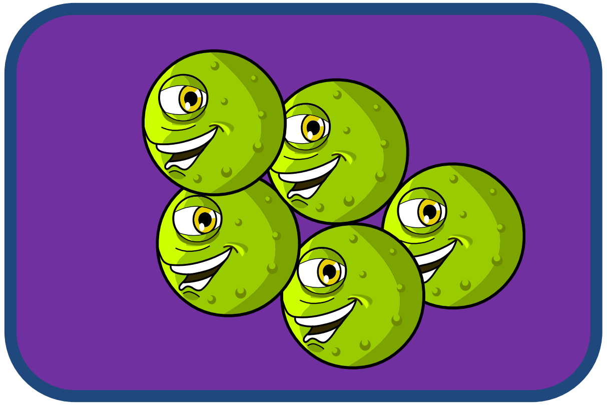
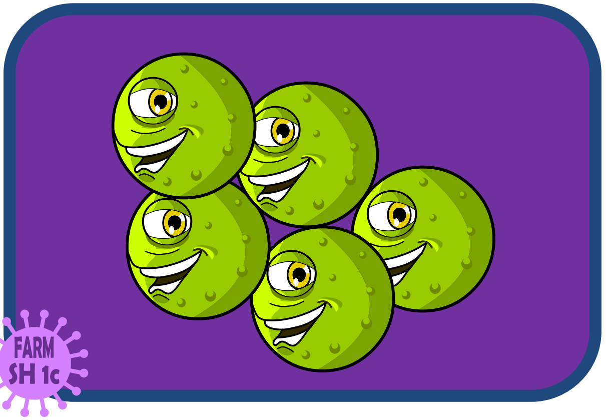
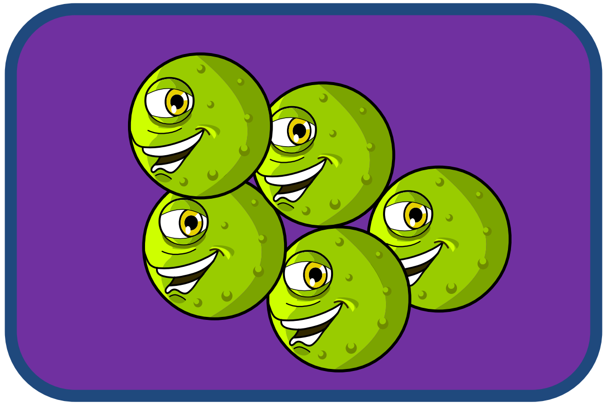
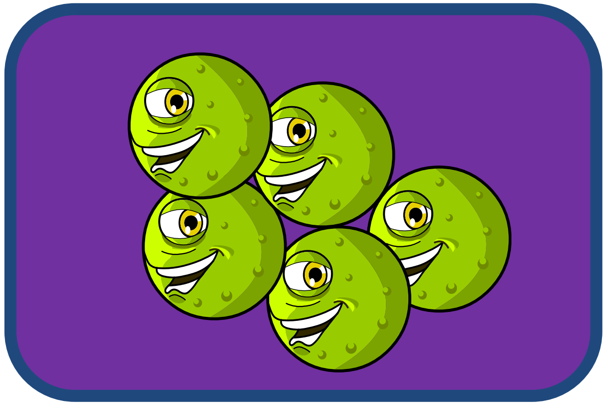

You washed away some harmful microbes when you washed your hands after playing with the animals.

Move on 3 spaces

You saw an animal scratching it's backside on a fence and stayed away from it because it might be covered in harmful microbes

Move on 5 spaces

You helped that farmer show that he uses useful microbes to make silage

Swap places with the person in the lead

The farmer thought that you were very clever because you knew that there were some useful microbes on the farm that help make crops grow

Move to on 4 spaces

You helped some smaller children wash their hands before they had their lunch

Move on 4 spaces

If you can name 1 place on the farm where there are useful microbes

Swap places with the person in front of you

One from: Compost bin, in the soil, in the dairy shed, in the silage tank, in the farm kitchen, root nodules

You rinsed off your wellie boots before you left the farm washing away any microbes that might be on them

Move on 1 space

If you can tell why we should wash our hands with soap and water and not just hand gel or wet wipes

Move on 8 spaces

Soap breaks up the oils on our hands that microbes stick to, and water rinses them away. Hand gel does not remove microbes from the hands, so soap and water is better.

If you can name 3 places on the farm where there might be harmful microbes

Swap places with the person in the lead

Three from: On an animal's body, in the animal's pen, in animal's poo, on a fence, on the ground where animals walk

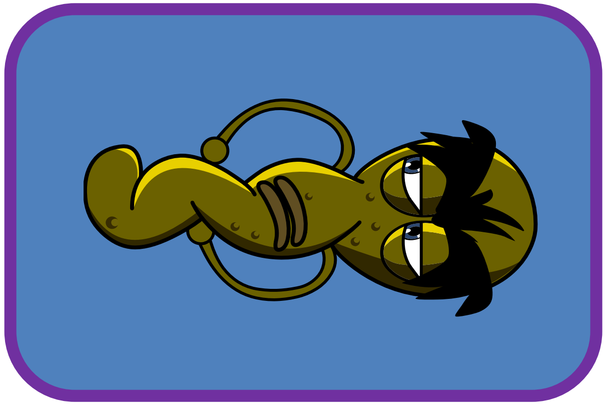
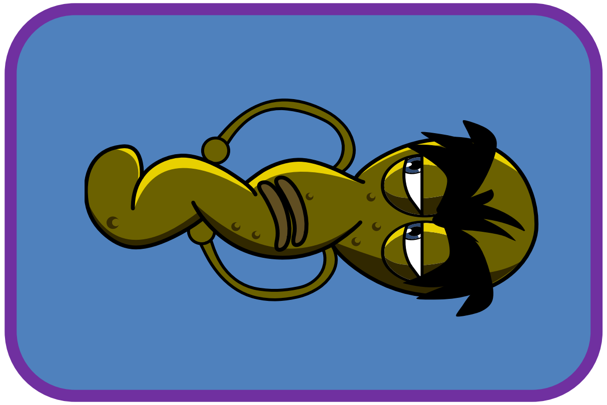
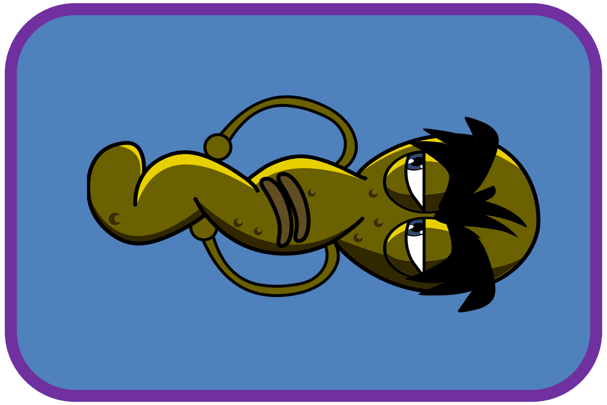
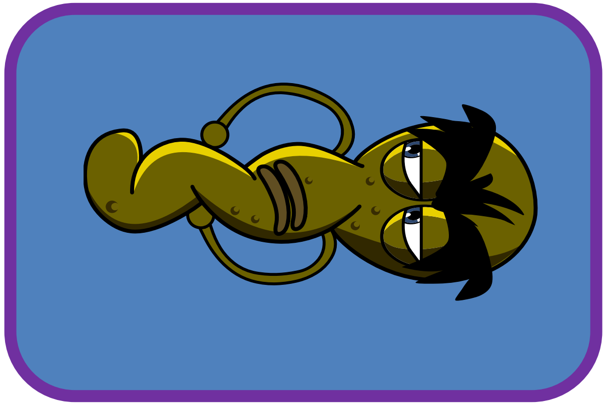
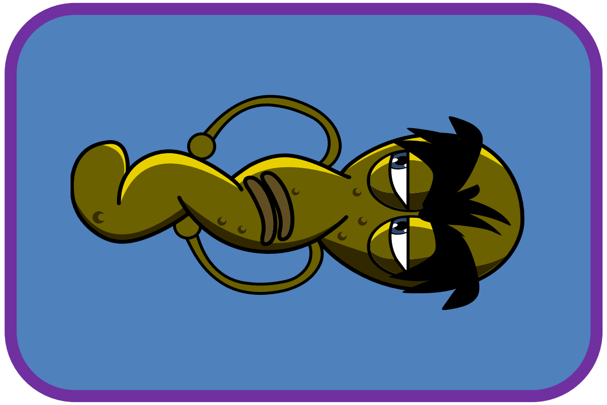
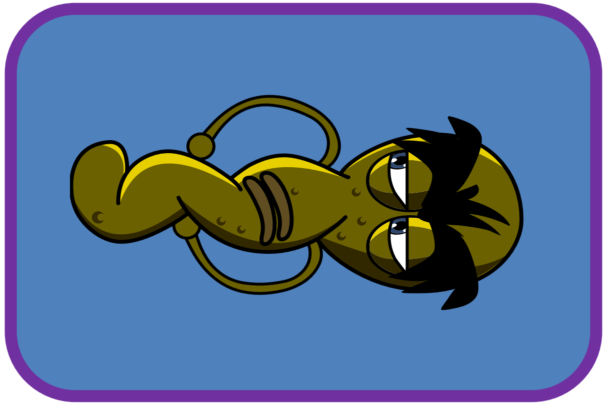
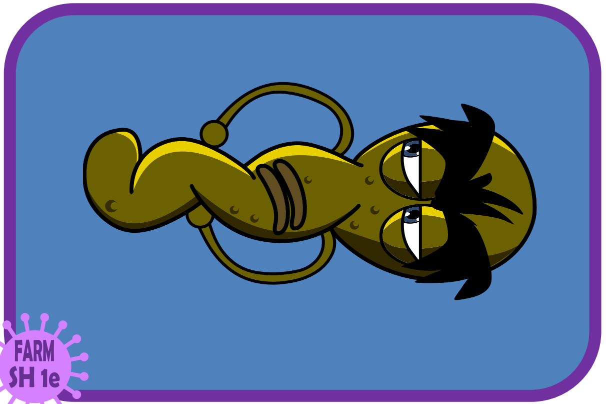
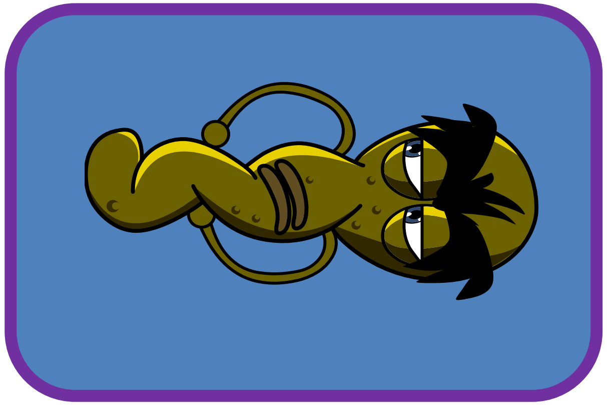
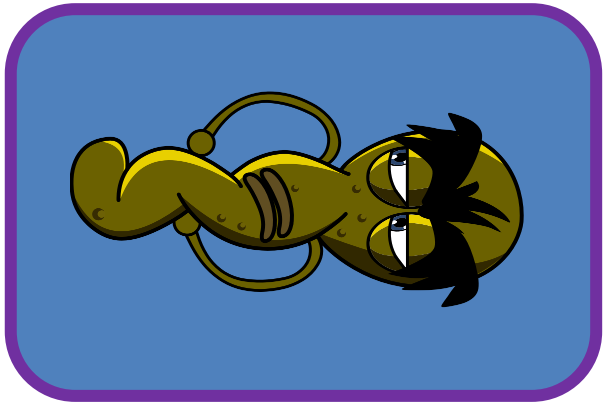

Move back 3 spaces

But stay where you are if you can answer the question

What should you do if you have tummy ache a couple of days after visiting the farm?

Stay off school and drink lots of fluids, and ask your parent to let your GP know that you were on a farm visit.

Swap places with the person in last place

But stay where you are if you can answer the question

Can harmful microbes be found on pets that live at home?

Yes, pets can carry harmful microbes that can make you unwell, so wash your hands after playing with them.

Swap places with the person behind you

But stay where you are if you can answer the question

Give an example of a harmful microbe that you can find on the farm?

Either: *E. Coli*, Salmonella, Campylobacter

Move back 5 spaces

But stay where you are if you can answer the question

Can you still eat your sandwich if you drop it on the ground whilst on the farm?

No, harmful microbes could have transferred to the sandwich from the ground. If you eat it, it may make you unwell.

Move back 4 spaces

But stay where you are if you can answer the question

Why is it better to wear wellies rather than trainers whilst visiting the farm?

Wellies are easier to rinse off at the end of the visit, and have less crevices where microbes can stick and hide.

Move back 1 space for every wrong answer

Name three things that you should not do whilst in the animal pen?

Three from: eat, suck your thumb, lick your fingers, touch animal faeces, sit on the ground, kiss the animals, touch your face

Move back to space number 2

But stay where you are if you can answer the question

Where on an animal's body is there likely to be a lot of harmful microbes?

Either: feet, mouth, rear end, tail

Move back 2 spaces

But stay where you are if you can answer the question

You see an apple that has fallen from the apple tree into the cow field, and you feel hungry. Why should you not eat it?

No, harmful microbes could have transferred to the apple from the ground. If you eat it, it may make you unwell.

Move back 1 space

But stay where you are if you can answer the question

Which is better – washing hands under running water from an outside tap, or in a bowl of water?

Running water – because it removes more harmful microbes and the water is cleaner.

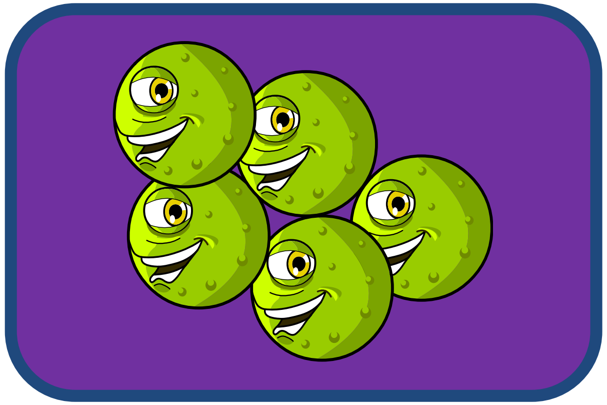
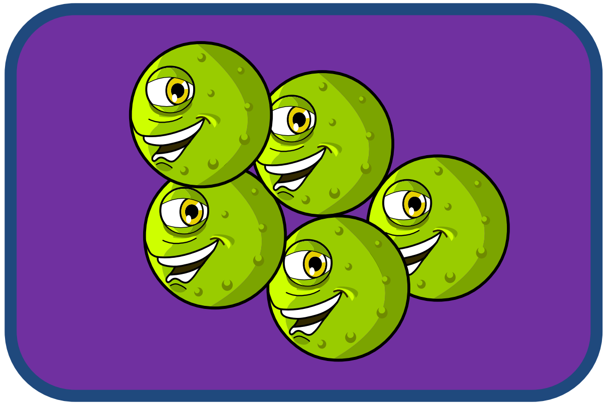
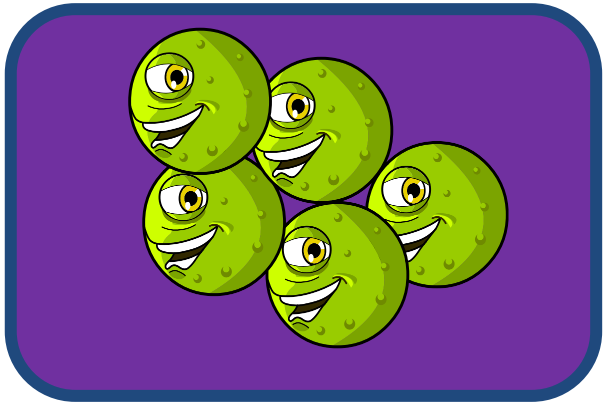
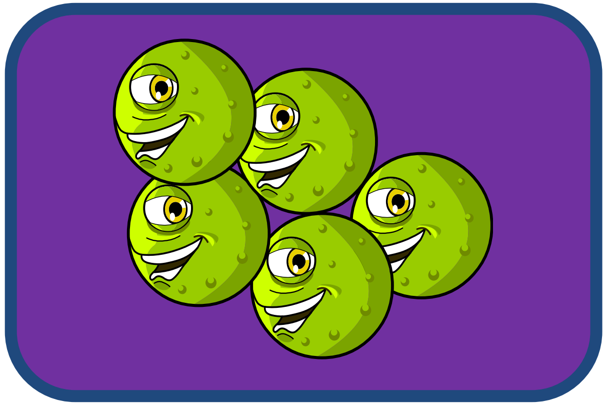
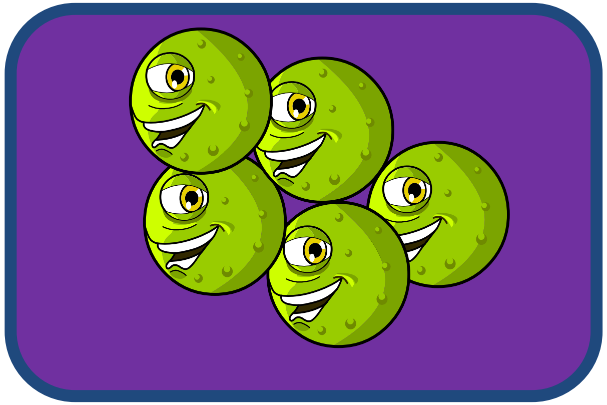
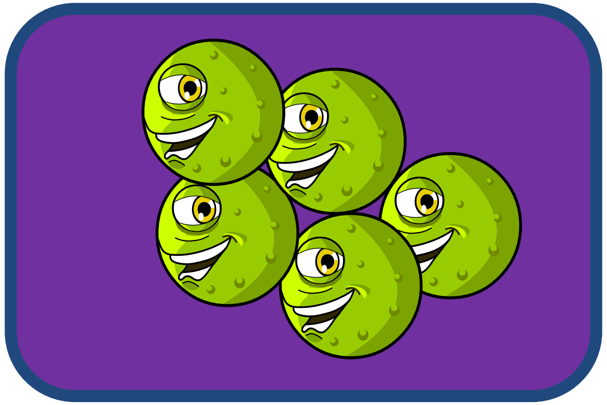
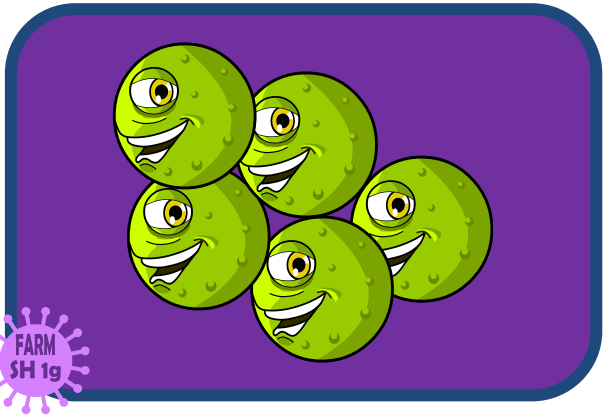
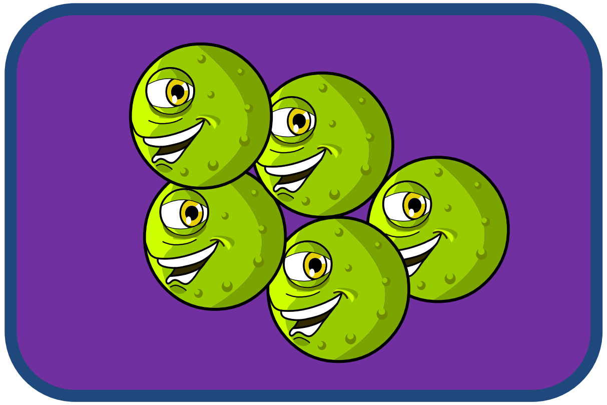
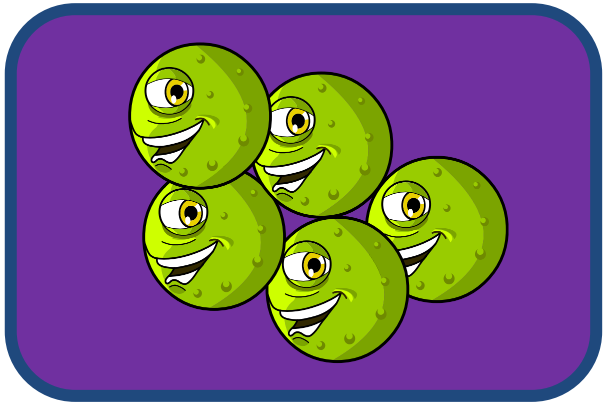

FARM SH 1g

You remembered to give your parents the information sheet from the farmer about what to do after the farm visit

Move on 3 spaces

You dropped a sweet on the ground and put it straight into the bin

Move on 5 spaces

You fell over and got muddy hands, so you washed them with soap and water before touching other people

Swap places with the person in the lead

You remembered to eat your lunch in the picnic area, and you washed your hands on the way

Move to the next even square

You remembered that animal poo can contain harmful microbes, so you avoided stepping in the cow pat!

Move on 4 spaces

There are more useful microbes than harmful microbes on the farm – true or false?

Swap places with the person in front of you

True – many of these microbes help the farmer to produce food for us.

You took off your shoes when you got home from the farm and left them in the porch so that an adult could clean them

Move on 1 space

If you can name a useful microbe that can be found on a farm

Move on 8 spaces

Either: Lactobacilli, Thermophiles, Rhizobia

If you can name a type of food or drink that is made with the help of a useful microbe on the farm

Swap places with the person in the lead

Either: yogurt, milk, bread, cereal or any other crop or vegetable, fruit cider, beer

FUN ON THE FARM

60	[illegible]	[illegible]	57	56	55	54	53	[illegible]	51
41	42	[illegible]	44	45	[illegible]	47	48	49	50
[illegible]	39	[illegible]	37	36	35	[illegible]	33	32	31
21	[illegible]	23	24	25	[illegible]	27	28	29	[illegible]
[illegible]	19	18	17	16	[illegible]	14	[illegible]	12	11
1	2	3	[illegible]	[illegible]	6	7	8	[illegible]	10

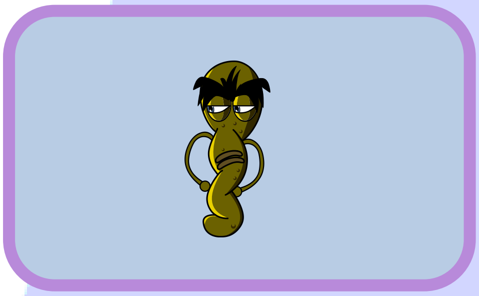

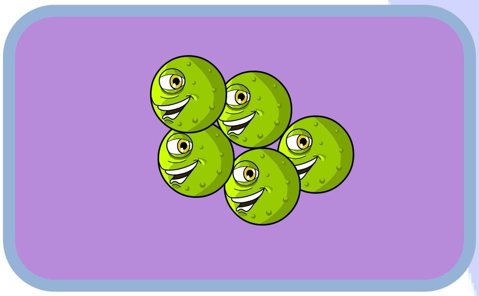

FUN ON THE FARM

BOARD GAME

e-Bug

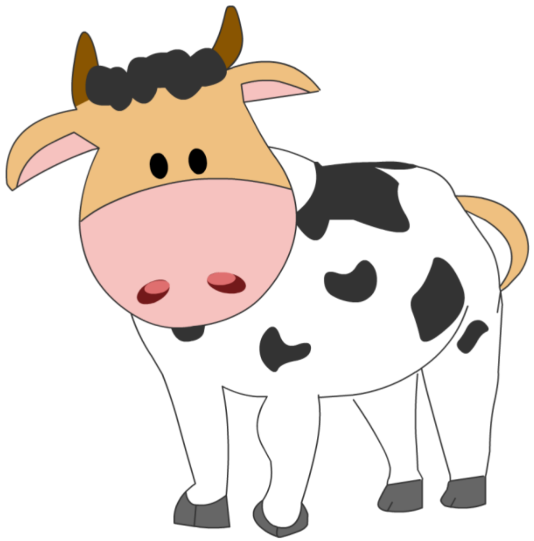

Where on the cow might you find lots of harmful microbes?

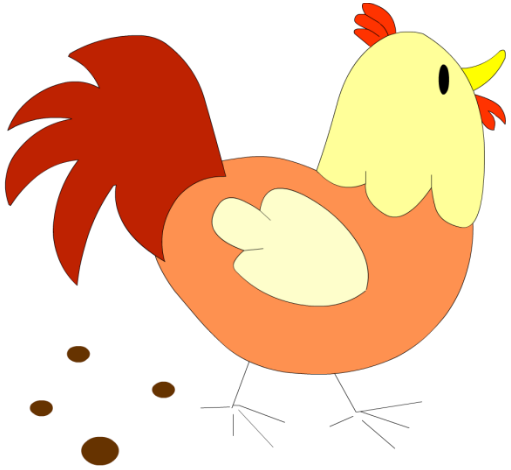

Why should you be careful to wash your hands after holding this chicken?

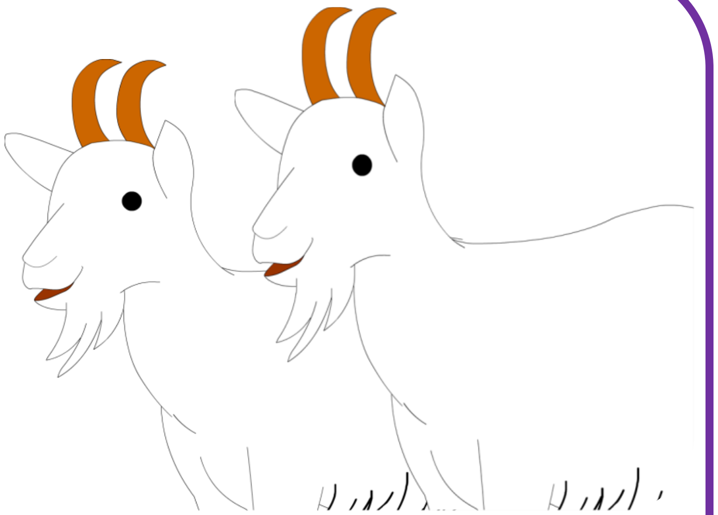

Where on the farm might these goats spread any microbes that they carry?

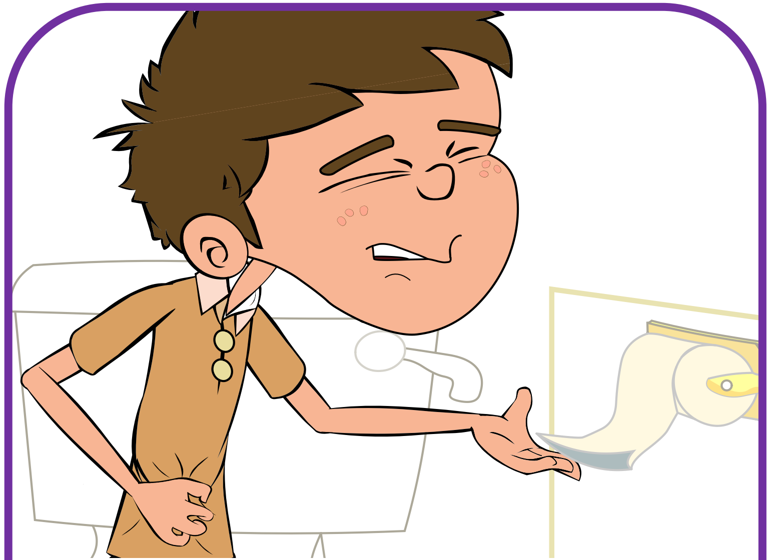

Harry caught a tummy bug after visiting a farm – do you know why?

FARM SW 1

Farm Hygiene
e-Bug Social Networking

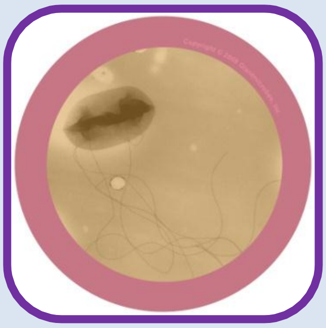

I'm Naughty unlike my good *E. coli* cousins... they can be helpful to humans but I'd much prefer to make them ill!

Friends

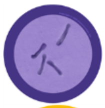

C. difficile

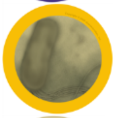

H. pylori

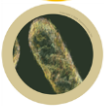

Shigella

Photos

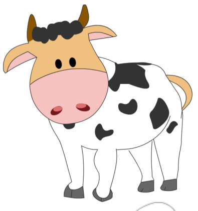

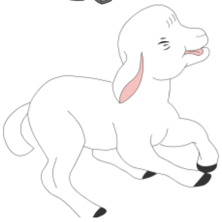

Profile

Escherichia coli 0157 (Esk-Er-Ic-E-A)

Nicknames: *E. coli*

Address: 1st Bend, Your Gut, The Human Body, N05E 2T0E

Alternative Address: 1 Goats Tongue, Cows Back Lane, Farm Hill, W00L 1EE

Likes: Making people sick (haha!), Animal droppings – Yum! I love to turn the human gut into a wild rapid ride that ends with a poo skydive down the loo... good times! Also, alcohol rub and hand wipes are my favourite, people think they are being so clever trying to wipe me away – little do they know that these things just won't cut it. I'm stronger than that you know!

Dislikes: Antibiotics... Urgh! And drinking lots of water (it makes my life harder, sigh.) I also HATE soap and water, I have to cling on to the skin SO much harder than if people just used water to rinse their hands.

The Wall

Steve the Sheep *E. coli*, I've been missing you! Ever since you left me to go off on holiday to that human body! Hope you are having fun causing trouble in their tummy! Baa x

Norovirus Hey, just saw an advertisement for the *Convention of Vomiting and Diarrhoea* this year... we will be able to learn new ways of making people sick! Hooray! See you there, N.

Doctor Alan *E. coli*, you have been banished from my patients bodies, thanks to my clever suggestion that they should drink lots of H_2O and stay away from other people until they get rid of you. Also the hand washing with soap worked a treat! Better luck next time!

Add as Friend?

Yes **No**

Farm Hygiene
e-Bug Social Networking

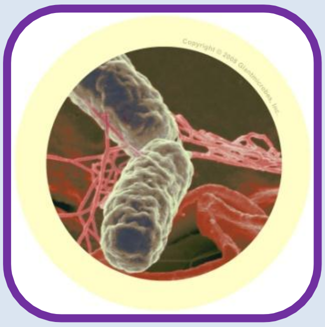

Profile

Salmonella (Sam-on-ella)

Job: I'm a proud member of the *Salmonella* Army – there are over 2500 different types of us!

Address: Stomach Hollow, The Human Body, V0M 1T5

Alternative Address: 2 Chicken Eggs, Nest Box, Field Green, 1CL UCK

Likes: Me and my *Salmonella* army pals like nothing more than to have a good old party down at the farm... they sure know how to make us welcome! But I also like to travel... I LOVE hot countries! So if you're planning on going anywhere soon you could pick me up whilst you're there!

Dislikes: Any people who wash their hands with soap... spoil my holidays won't you!

My main aim in life is to settle down – but that never seems to happen! I can't help it if I'm always causing food poisoning, can I?!

Friends

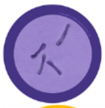
C. difficile

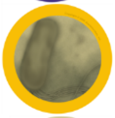
H. pylori

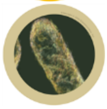
Shigella

Photos

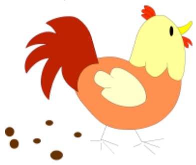

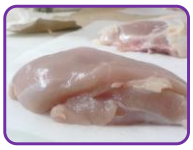

The Wall

Travel Operator Bob This is a quick message to say thank you for travelling overseas with us! With your help, together we are really reaching every corner of the globe! Next time I hope you bring your friends!

Daisy Duck *Salmonella*, will you just leave my eggs alone!! Everyone's stopped buying them since you came along and I really need the money – I've got my eye on a new pond... and you're ruining my plans!!!

Doctor Alan Oh I see – animals' guts aren't enough for you, you even want to conquer beansprouts to satisfy your infectious cravings! Don't worry, I'm preparing to ensure all my patients wash and cook all of their raw food carefully to spite you!

Add as Friend?

Yes **No**

Farm Hygiene
e-Bug Social Networking

Profile

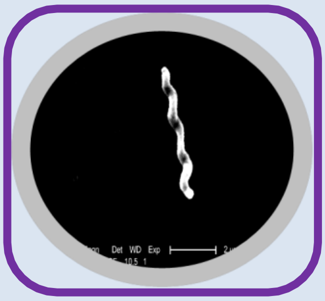

My speciality is causing bloody diarrhoea and painful stomach cramps... oh yeah, there's always fun when I'm around!

Campylobacter (Cam-pie-low-back-ter)

Address: Round Stomach Lane, The Human Body, PA1N FUL

Alternative Address: 1 Under Cooked, Poultry Lane, Market Farm, M3AT RAW

Likes: Playing gut twister! Although, even if I do say so myself, I'm pretty good at cramping up your insides! I also like chicken and unpasteurised milk... they are pretty cool places to hang. Got any raw meat in your fridge? I'm not fussy, if it is sitting around, I'll make it my home.

Dislikes: Well cooked meat and high oven temperatures... they are the enemy!

Friends

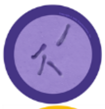 *C. difficile*

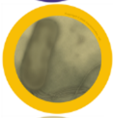 *H. pylori*

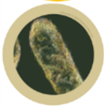 *Shigella*

Photos

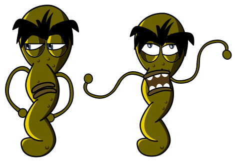

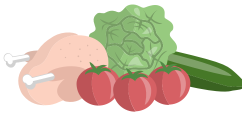

The Wall

Patient Amy You are horrible *Campylobacter*, you've made me SO ill! I wish I had never made friends with you!

Kevin the Chicken Hi mate, just you go careful and wear sun screen when you visit that kitchen this week – you might die if the temperature in that oven is too high!

Doctor Alan *Campylobacter*... I'm on to you! I know you hide around and can spread to other people even after my patients start feeling better. But you better watch out... I've been warning them about the benefits of washing hands with soap and water!

Add as Friend?

Yes No

Farm Hygiene
e-Bug Social Networking

Group

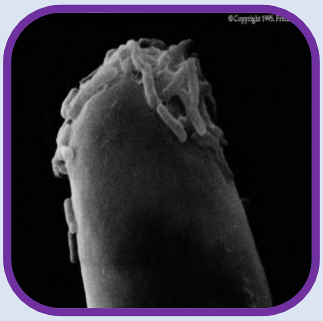

Rhizobia (Rye-zo-bee-a)

Nicknames: Nitrogen fixing bacteria

Address: Roots Range, Legume Lane, Under Earth Village, N1TR OG3N

Likes: We are 'stay at home' kind of microbes, once we find a nice spot on a root, we hang out there all day and night! Not that we are lazy though – we pay our way by filling the soil with loads of ammonia that plants need to grow, using natural gas that is in the air!! Sounds like magic, but it's all about the tricks of the trade... and it keeps us with a roof over our heads!

Dislikes: Too much oxygen – I know it sounds silly to you humans but we just don't like it, it messes up our important work.

Rhizobia are great at one thing – changing nitrogen gas into a healthy snack for plants. But we don't do this alone; we live inside bean, clover and pea plants that help us with our difficult iob!

Friends

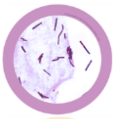
L. acidophilus

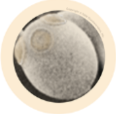
S. cerevisiae

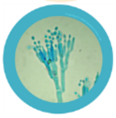
Penicillium

Photos

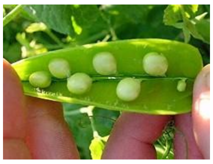

The Wall

Clive the Clover Hey, how you doing down there? There's a load of seeds on their way that are being really fussy, so we need to work extra hard to make the soil just right for them! We can't do this alone, you and me need to work together to get this right!

Farmer David Hi guys, thought you might like to know – I just won an award for my fantastic cereal crop at the town hall. I just wanted to say, I couldn't have done it without you – thanks very much!

Subscribe to group?

Yes **No**

Farm Hygiene
e-Bug Social Networking

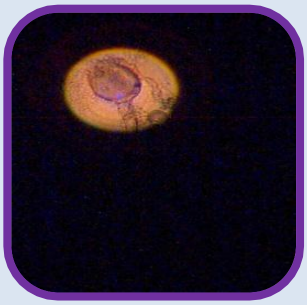

Group

Thermophiles (Therm-oh-fi-alls)

Nicknames: a big group of heat lovers!

Address: Peat Bog, Compost Bin, Round Barn Yard, TOAS T1E

Alternative Address: Cereal Lane, Over Soil Spread, Field Green, 1MEA DOW

Likes: We LOVE a good old pile of plant clippings – mix in some cut grass and soil and that makes a fabulous meal! To be a member of the thermophile gang, you can be a bacterium or a fungus – it's all about working in high temperatures! Decaying plants are our specialty – we break them down and recycle them right back into yummy yummy nutrients, all in the comfort of tropical heat!

Dislikes: You know we like heat – but we hate the cold! And when we say cold we mean 20ºC - at those temperatures the bacteria among us won't even get out of their nice protective endospores! Brrr!

We are thermophiles because we love the heat! Take a trip to the middle of some compost and you will find it is lovely and warm – which is perfect for us – it just makes us work harder!

Friends

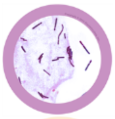
L. acidophilus

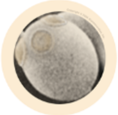
S. cerevisiae

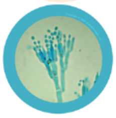
Penicillium

Photos

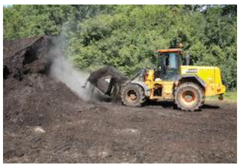

The Wall

Sally Strawberry Hi thermophiles, thanks for my tasty dinner that you created in the compost kitchen, it was fantastic! I'll be asking Farmer David for some more next week, so keep going! X

Lactobacillius Dear all, I hope you will be coming to my party next week – it's a field party to celebrate another year of great food production on the farm. I just hope it doesn't get gate crashed – I don't want any nasty bacteria to come!

Eco Eddie Congratulations thermophiles, you have been nominated for an eco-award for all your recycling efforts. By turning all that plant waste into nutrients for the farm, you really help the ecosystem. Well done!

Subscribe to group?

Yes **No**

Farm Hygiene
e-Bug Social Networking

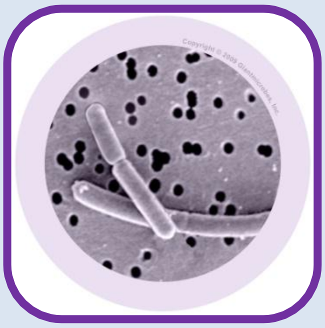

Group *Lactobacilli* (Lac-Toe-Ba-Sil-i)

We like a good boxing match when we are hanging out in the human gut, so watch out all you harmful bacteria – we fight for the good health of humans! Ding Ding, round one coming up!

Nicknames: Probiotic bacteria

Address: Swimming Inn, Cow's Milk Pond, Dairyfresh Farm,
F3R M3NT

Alternative Address: 1. Useful Microbe, Small Intestine Corner, Tummy Place, PPL5 GUT

Likes: Our favourite food is lactose – man that milk sugar is hard to beat!! It gives us bags of energy, and we know it makes everyone happy when the result of our fermentation is a lovely smooth yogurt fit for a king! Also, some of us are great at making silage – fermented grass that cows and sheep can eat all year round... they love it!

Dislikes: Nasty microbes that are horrible to humans – what's up with that? Humans and *Lactobacilli* have always been bessie mates – we help each other out, right?!

Friends

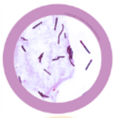
L. acidophilus

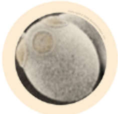
S. cerevisiae

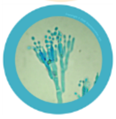
Penicillium

Photos

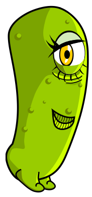

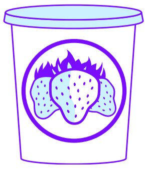

The Wall

Society for Dairy Products Dear *Lactobacilli* members, we are holding a lesson on the importance of bacterial cultures to the food supply, and were hoping you could do a presentation? You really are some of the best at this sort of thing! Please get back to us soon.

Patient Amy Thanks so much all you friendly *Lactobacilli*, after *Campylobacter* came to stay, you really helped me get back on my feet and feel healthy again. Love Amy x

Dorothy the Cow Keep it up *Lactobacilli*! Because of all your help, my milk is really popular and is selling out fast! Soon I'll be able to afford that holiday to the beach I've always wanted... I can't wait to feel the sand beneath my hooves!

Subscribe to group?

Yes **No**

e-Bug

Farm Hygiene
Game Rules

Fun on the Farm!

The Rules

1. Roll the die to see which player goes first – highest roll gets it!
2. Take turns in a clockwise order to roll the die and move along the board.
3. If you land on a microbe, the person to your left must pick up a card from the pack and ask you the question.
4. Whoever gets to the end of the board first wins!

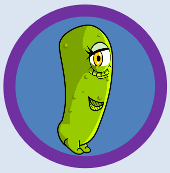

Select your counter

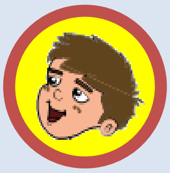

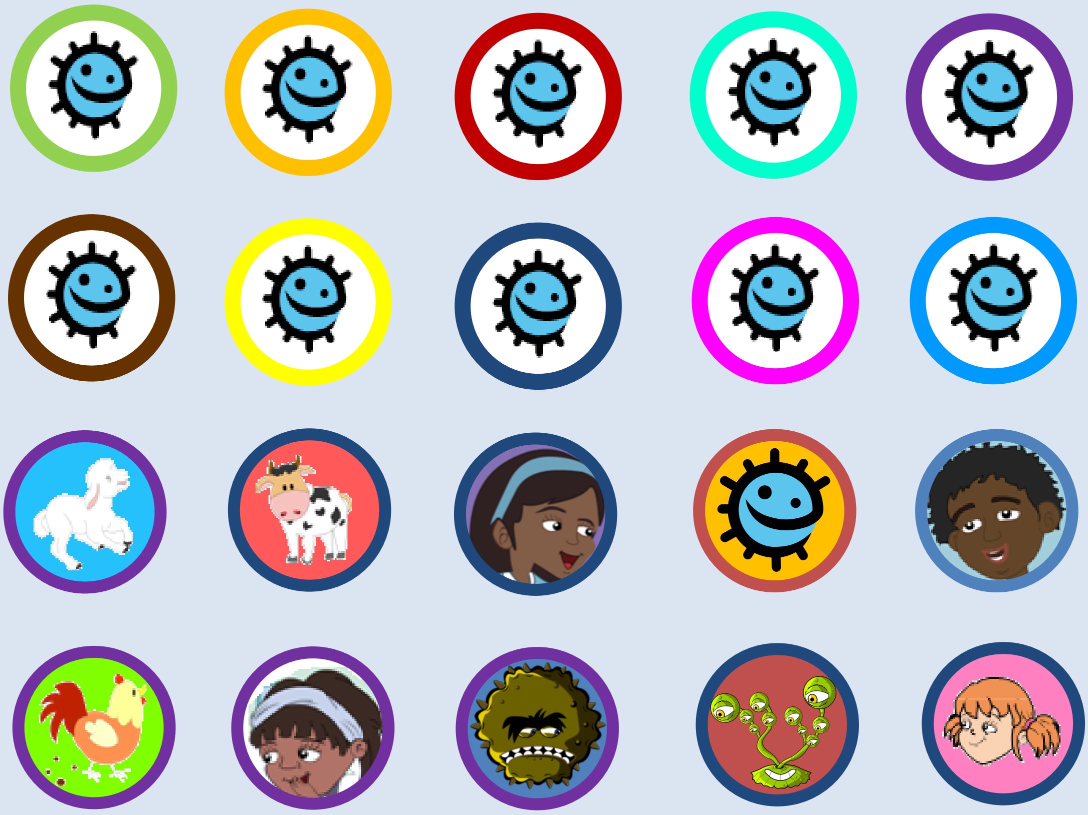

Supplement: File S1 — e-Bug Fun on the Farm Lesson Plan. (PDF) [file pone.0075641.s001.pdf]
