# Supplementary material for: Fun on the Farm: Evaluation of a Lesson to Teach Students about the Spread of Infection on School Farm Visits
Source: PLoS One. 2013 Oct 16;8(10):e75641. doi: 10.1371/journal.pone.0075641 (PMC3797722; doi:10.1371/journal.pone.0075641)
Supplement: File S2 — Pre- and Post- Intervention Student Knowledge Questionnaire. (PDF) [file pone.0075641.s002.pdf]

School Name: **Student Name:** **Questionnaire 1**

Introduction to microbes		AGREE	DISAGREE	DON'T KNOW
If you cannot see a microbe it is not there				
All bacteria are harmful				
Bacteria and Viruses are the same				
Fungi are microbes				
Microbes are found:	in boiling water			
	in our mouths			
	on our hands			
	on animals			

Hand hygiene		AGREE	DISAGREE	DON'T KNOW
Bad microbes can spread:	when you touch someone's hands			
When visiting a farm people should wash their hands:	before eating			
	After petting the animal			
	After eating			
	after touching the crops			
If people wash their hands they are less likely to get ill				
Washing with soap **and** water removes more microbes than water alone				
Washing hands with alcohol gel / wipes will remove all bad microbes on the farm				
Using alcohol hand gel is better than washing hands with hot running water and soap				

Specific Farm Questions		AGREE	DISAGREE	DON'T KNOW
At the farm, microbes are found:	On cows			
	On gates			
	In the grass			
	On your wellie boots			
Some microbes can help crops to grow				
There are more useful microbes on the farm than harmful ones				
There is no need to wash your hands after stroking a farm animal				
Washing hands is the best way to stop the spread of harmful microbes				
You cannot pick up bad microbes from kissing or hugging an animal on the farm				
It is OK to eat your sweets while walking around a farm				
We should wash our wellie boots before we leave the farm				
We should eat our packed lunch at the farm picnic tables				
